# The epitaxy of 2D materials growth

**DOI:** 10.1038/s41467-020-19752-3

**Published:** 2020-11-17

**Authors:** Jichen Dong, Leining Zhang, Xinyue Dai, Feng Ding

**Affiliations:** 1 Centre for Multidimensional Carbon Materials, Institute for Basic Science, Ulsan, 44919 Korea; 2 School of Materials Science and Engineering, Ulsan National Institute of Science and Technology, Ulsan, 44919 Korea; 3 School of Materials Science and Engineering, Shandong University, 250061 Jinan, China

**Keywords:** Two-dimensional materials, Synthesis and processing, Surfaces, interfaces and thin films

## Abstract

Two dimensional (2D) materials consist of one to a few atomic layers, where the intra-layer atoms are chemically bonded and the atomic layers are weakly bonded. The high bonding anisotropicity in 2D materials make their growth on a substrate substantially different from the conventional thin film growth. Here, we proposed a general theoretical framework for the epitaxial growth of a 2D material on an arbitrary substrate. Our extensive density functional theory (DFT) calculations show that the propagating edge of a 2D material tends to align along a high symmetry direction of the substrate and, as a conclusion, the interplay between the symmetries of the 2D material and the substrate plays a critical role in the epitaxial growth of the 2D material. Based on our results, we have outlined that orientational uniformity of 2D material islands on a substrate can be realized only if the symmetry group of the substrate is a subgroup of that of the 2D material. Our predictions are in perfect agreement with most experimental observations on 2D materials’ growth on various substrates known up to now. We believe that this general guideline will lead to the large-scale synthesis of wafer-scale single crystals of various 2D materials in the near future.

## Abstract (web summary)

Advances in our ability to manipulate genetics leads to deeper understanding of biological systems. In this perspective, the authors argue that synthetic genomics facilitates complex modifications that open up new areas of research.

## Introduction

Two-dimensional (2D) materials are potentially the most promising materials for future device applications but in practice, wafer-scale single crystals of various 2D materials are needed to realize these applications^1–4^. Recently, the seamless coalescence of millions of well-aligned islands of a 2D material epitaxially grown on a substrate has been successfully used to synthesize wafer-scale single crystals of graphene^5–7^, hexagonal boron nitride^8,9^, and MoS_2_^10^. This strategy is expected to be generalized to grow various 2D single crystals in the near future. Nevertheless, the unique behavior of 2D materials growth, different from that predicted by classical theory of epitaxy, necessitates the development of a general theory for the epitaxial growth of 2D materials^5–22^.

In graphene CVD growth, the zigzag (ZZ) edge is generally the slowest propagating edge because of its highest barrier for edge propagation^23,24^ and the alignment of graphene on a substrate has been broadly observed to be dependent on the symmetry of the substrate. For example, graphene islands grown on a Cu(111) or Cu(110) surface are well-aligned but those grown on a Cu(100) surface are observed to be along two perpendicular directions^5,11,12^. Among the three low-index Cu surfaces, highly robust alignment of graphene can be obtained on the Cu(111) surface and currently, the principal method of graphene single crystal production is by epitaxially growing graphene single crystals on Cu(111) surface^5–7^.

Because of the lower C_3V_ symmetry of hBN, the alignment of hBN on a substrate is different from that of graphene. Three of its six ZZ edges are nitrogen terminated and the other three are boron terminated (named as ZZN and ZZB edges hereafter). In most experiments, the ZZN edge has been proven to be the slowest propagating and kinetic Wulff construction leads to triangular hBN islands enclosed by three ZZN edges^25–27^. In contrast to epitaxial graphene growth, well-aligned hBN islands have rarely been observed. When grown on Cu(111) or Cu(110) surfaces, triangular hBN islands aligned along two opposite directions were found^13–15,28^, while those grown on Cu(100) surfaces had four different orientations^14^. Recent works have shown that well-aligned hBN islands can be successfully achieved by using a Cu substrate with tailored step edges, thus enabling epitaxial growth of wafer-scale hBN single crystals^8,9^.

Similar to hBN, most transition metal dichalcogenides (TMDCs) possess three-fold symmetry and present very similar epitaxial behavior on substrates; for example, with two possible alignments on Au(111)^16^, Al_2_O_3_(0001)^17–19^, and GaN(0001)^20^ surfaces. Well-aligned WS_2_ islands have been grown on hBN surface^21^ and nearly well-aligned WSe_2_ islands have been grown on a vicinal Al_2_O_3_(0001) surface^22^. Most recently, centimeter scale single-crystalline MoS_2_ was obtained by the coalescence of well-aligned MoS_2_ grains on a vicinal Au(111) surface^10^.

All these experimental observations strongly indicate that the alignment of a 2D material on a substrate depends on both its symmetry and that of the substrate and a general theory for 2D materials epitaxy that helps to predict the alignment of various 2D materials on different substrates is highly desirable to serve as a guideline for experimental design.

Here, based on extensive density functional theory (DFT) calculations, we present a general theory to explain how the alignment of a 2D material on a substrate is intimately related to its interaction with the substrate and how the epitaxial growth of the 2D material on a substrate is critically dependent on the interplay of the symmetries of the 2D material and the substrate. Our theory explains most known experimental observations on 2D material epitaxial growth and hence can serve as a guideline for the experimental synthesis of various 2D single crystals, as well as 2D polycrystalline materials with designed grain boundaries.

## Results

### 2D material–substrate interaction and the alignment of a 2D material on a substrate

There are hundreds of important 2D materials and the possible substrate types are also of the same order of magnitude. So, it is impossible to calculate the interactions of all possible combinations of 2D materials and substrates, which is greater than 100,000. Without losing the generality, we can classify the interactions between 2D materials and various substrates into two sceneries:

- (i) The edge of the 2D material is terminated by the substrate, such as graphene or hBN on an active metal substrate, where the strong interaction between the edge of the 2D material and the pristine substrate facet determines the alignment of the 2D material and its epitaxial growth behavior^29,30^;
- (ii) The edge of the 2D material is self-passivated or terminated by active atoms from the environment of its growth, such as H or OH groups^31–34^, where the weak interaction between the bulk of the 2D material and the pristine substrate facet dominates the alignment of the 2D material.

**Fig. 1:**
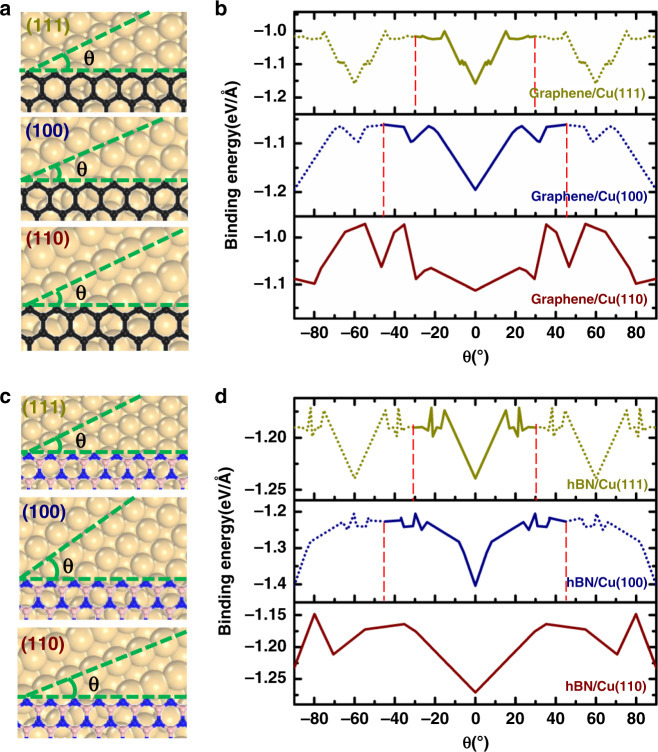
Binding energy of ZZ(ZZN) edge of graphene(hBN) on three low-index Cu surfaces. Model of a graphene ZZ edge on three low-index Cu surfaces **a** and the calculated binding energies of the edge on the three substrates as a function of the alignment angle **b**. Model of a ZZN edge of hBN on the three low-index Cu surfaces **c** and the calculated binding energies of the edge on the three substrates as a function of the alignment angle **d**. Vertical lines highlight the periodicity of the binding energy curves.

To establish an epitaxial relationship between a 2D material and a substrate for scenery (i), we firstly explore the interaction between the edges of graphene or hBN with the three low-index Cu surfaces, namely Cu(111), Cu(100), and Cu(110). The calculated binding energies between a graphene (hBN) ZZ(ZZN) edge on the three low-index Cu surfaces as a function of the angle of edge alignment that is defined as the angle between the edge and a Cu〈110〉 direction of the substrate, are shown in Fig. 1 (please refer to method section and Supplementary Figs. 1–6 in Section 1 of supplementary information (SI) for more details on the calculation and modeling). We clearly see that on each of the three low-index surfaces, the strongest binding energy appears when the ZZ(ZZN) edge of graphene (hBN) is along a Cu〈110〉 direction of the surface; the difference between the binding energy minimum and maximum is significant, >0.2 eV per edge atom. Hence, on a Cu surface, a well-aligned small graphene or hBN island of ~2 nm (which has only ~200 atoms of which ~40 are at the edge) has an energy advantage of >8 eV over misaligned ones. This binding energy difference is large enough to maintain a growing graphene or hBN island in a well-aligned configuration on a Cu surface.

**Fig. 2:**
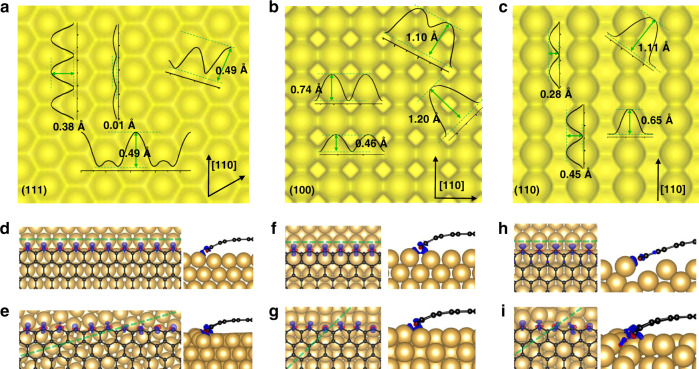
Charge density analysis of low-index Cu surfaces and zigzag edges of graphene adsorbed on them with different orientations. **a–c** The electron density profiles with isovalue of 0.03 Bohr^−3^ of Cu(111), Cu(100), and Cu(110) surfaces, respectively. The height of the isosurface along different directions of the different Cu surfaces are shown. The Cu[110] directions on these surfaces are also marked. **d**–**i** Charge density differences with isovalue of 0.01 Bohr^−3^ of graphene on Cu(111)/Cu(100/Cu(110)) surfaces with misorientation angles of 0^o^ **d**, **f**, **h** and 15.3^o^/45^o^/35.3^o^ **e**, **g**, **i**. Blue and red colors in **d–i** represent electron accumulation and depletion, respectively.

To elucidate the reason behind the strongest binding of a ZZ(ZZN) edge along the 〈110〉 direction on a Cu surface, we have plotted the electron density distributions of Cu(111), Cu(100), and Cu(110) surfaces, respectively, in Fig. 2a–c. It can be seen that on all the three surfaces, the isosurface fluctuation in electron density is the lowest along the 〈110〉 direction, indicating that the close-packed 〈110〉 atomic rows form a pattern with alternative ridges and valleys of uniform height on the surface. In a straight ZZ(ZZN) edge of graphene(hBN), the less stable edge atoms form a straight line and this straight edge is preferentially passivated by either a ridge or a valley of the Cu surface instead of crossing over ridges and valleys on the surface, which results in distortion of the edge. To further illustrate preferential passivating of the graphene ZZ edge by a close-packed atomic row, we compare the atomic structures of the interfaces and the charge density differences of the graphene edge along both 〈110〉 and other directions on the three low-index Cu surfaces (Fig. 2d–i). When the graphene ZZ edge is aligned along the Cu〈110〉 direction, all the edge atoms are well passivated by a Cu〈110〉 atomic row and the edge remains straight. In contrast, if the graphene edge is along another direction, some of the edge atoms are poorly passivated and the edge is no longer straight because of the fluctuating ridge-valley pattern of the surface. The above analysis clearly shows the superiority of the close packed direction of a substrate in passivating a high-symmetric edge in a 2D material.

Our analysis of a straight graphene/hBN edge preferring the direction of valley or ridge of the isosurface of Cu substrate can be generally applied to most combinations of 2D materials on various substrates. It is noted that the lattice constant of graphene/hBN ZZ edge matches that of the 〈110〉 direction of Cu substrate well. Thus, it is worth to consider a system without perfect lattice-match. To address the effect of lattice-mismatch, we consider the interaction between a ZZ edge of graphene and the Pt(111) surface, where the lattice constant of graphene ZZ edge is about 12.6% smaller than that of Pt〈110〉 direction. The binding energies of a graphene ZZ edge on the Pt(111) surface as a function of the alignment angle of the graphene edge are shown in Supplementary Fig. 7. As expected and similar to that of a graphene ZZ edge on Cu(111) surface, the graphene ZZ edge prefers the alignment along a 〈110〉 direction of the Pt(111) surface. As an example, above calculation suggests that the conclusion that the slowest propagating (also high-symmetric) edge of a 2D material prefers to align along the high-symmetric direction of an active metal substrate, regardless of the lattice-match between the 2D material and the substrate.

**Fig. 3:**
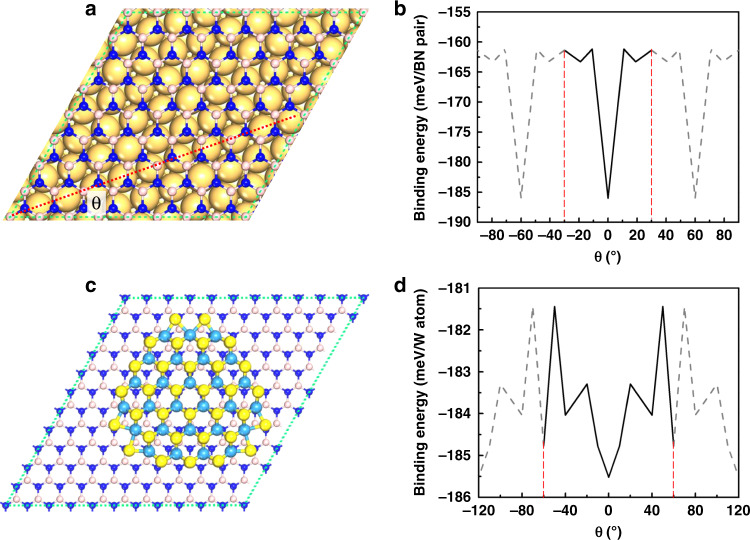
The calculated interactions between the bulks of 2D materials and substrates. Periodic boundary condition model of hBN on the Cu(111) surface **a** and the calculated binding energies of hBN on the Cu(111) surface as a function of the alignment angle (*θ*) between the ZZ direction of hBN lattice and a 〈110〉 direction of the Cu(111) surface **b**. Cluster model of a WS_2_ cluster on the hBN surface **c** and the calculated binding energies of the WS_2_ cluster on the hBN surface as a function of the alignment angle (*θ*) between the ZZ direction of WS_2_ and the ZZ direction of the hBN surface **d**.

If the edge of a 2D material is self-terminated, such as the selenium-terminated edges of TMDC materials^33^, or terminated by the H or OH functional groups, such as the edges of graphene or hBN grown on a less active metal substrate^31,32,34^. In such a scenery, the interaction between the 2D material edge and the substrate is no longer very strong and the dominating interaction is the weak interaction between the bulk of the 2D material and the substrate. To elucidate the alignment of a 2D material on a substrate for scenery (ii), we calculate the interaction between hBN and Cu(111) (Fig. 3a, b, and Supplementary Fig. 8) and Au(111) (Supplementary Fig. 9) surfaces, respectively, by using the periodic boundary condition models and the interaction between a triangular WS_2_ cluster and the hBN surface (Fig. 3c, d and Supplementary Fig. 10). All these calculations, together with previous studies of graphene on Cu(111) surface^35^ and MoS_2_ on Al_2_O_3_(0001) surface^36^, show that a high-symmetric direction of a 2D material (ZZ directions of graphene, hBN and TMDCs) prefers to align along the high-symmetric directions of a substrate, such as the 〈110〉 directions of Cu(111) and Au(111) surfaces, and 〈11$\bar 20$〉 direction of hBN and Al_2_O_3_ (0001) surfaces.

The above results allow us to draw a conclusion of the alignment of a 2D material on an arbitrary surface, i.e., a high-symmetric direction of the 2D island prefers to align along a high-symmetric direction of the substrate. Although we just explored a very limited systems of 2D materials grown on various substrates, as will be seen later, this rule is in accordance with most experimental observations on the epitaxial growth of various 2D materials, such as the CVD synthesis of graphene, hBN, and TMDCs on various transition metals or nonmetallic substrates (please refer to Section 2 in SI). Thus, we believe that this rule can be applied for the epitaxial growth of various 2D materials on different substrates.

### The alignment of 2D materials on various substrates

Having established the principle that determines the alignment of a 2D material on a substrate, we proceed to discuss the interplay between the symmetry of a 2D material and that of the substrate in epitaxial growth. Let us consider a 2D material with a G_2D_ symmetry group on a substrate with a symmetry group of G_sub_. The symmetry group of the whole system, G_2D@Sub_, must be a subgroup of either G_2D_ or G_Sub_ because any symmetry operation of G_2D@Sub_ will not change the alignment of the 2D material or the substrate. As shown in SI, we have proved that the number of equivalent but different directions of a 2D material on a substrate can be calculated by

1 $$N_1 = \frac{{\left| {{\mathrm{G}}_{{\mathrm{sub}}}} \right|}}{{\left| {{\mathrm{G}}_{2{\mathrm{D}}@{\mathrm{sub}}}} \right|}},$$

where $\left| {{\mathrm{G}}_{{\mathrm{sub}}}} \right|$ and $\left| {{\mathrm{G}}_{2{\mathrm{D}}@{\mathrm{sub}}}} \right|$ are the orders, or numbers of different symmetry operations, of G_sub_ and G_2D@sub_, respectively.

According to the principle of 2D materials alignment discussed in the previous paragraph, the presence of a high-symmetry edge of a 2D material along a high-symmetry direction of the substrate ensures that the symmetry group of the whole system, G_2D@Sub_, is the largest subgroup of both G_sub_ and G_2D_. We have considered various combinations of the symmetries of the 2D material and the substrate and the numbers of equivalent but different alignments of various 2D materials are shown in Table 1.

**Table 1:** The number of equivalent but different orientations of a 2D material on a substrate based on the interplay between their symmetries.

G_2D_	C_6V_				C_4V_				C_3V_				C_2V_			
G_Sub_	C_6V_	C_4V_	C_3V_	C_2V_	C_6V_	C_4V_	C_3V_	C_2V_	C_6V_	C_4V_	C_3V_	C_2V_	C_6V_	C_4V_	C_3V_	C_2V_
G_2D@Sub_	C_6V_	C_2V_	C_3V_	C_2V_	C_2V_	C_4V_	C_V_	C_2V_	C_3V_	C_V_	C_3V_	C_V_	C_2V_	C_2V_	C_V_	C_2V_
∣G_sub_∣	12	8	6	4	12	8	6	4	12	8	6	4	12	8	6	4
∣G_2D@sub_∣	12	4	6	4	4	8	2	4	6	2	6	2	4	4	2	4
*N*_1_	1	2	1	1	3	1	3	1	2	4	1	2	3	2	3	1

Here, G_Sub_, G_2D_ and G_2D@Sub_ are symmetry groups of the substrate, the 2D material and the 2D material–substrate system, ∣G_sub_∣ and ∣G_2D@sub_∣ are the orders of G_Sub_ and G_2D@Sub_; and *N*_1_ = ∣G_sub_∣/∣G_2D@sub_∣ is the number of equivalent but different directions of the 2D material on the substrate.

**Fig. 4:**
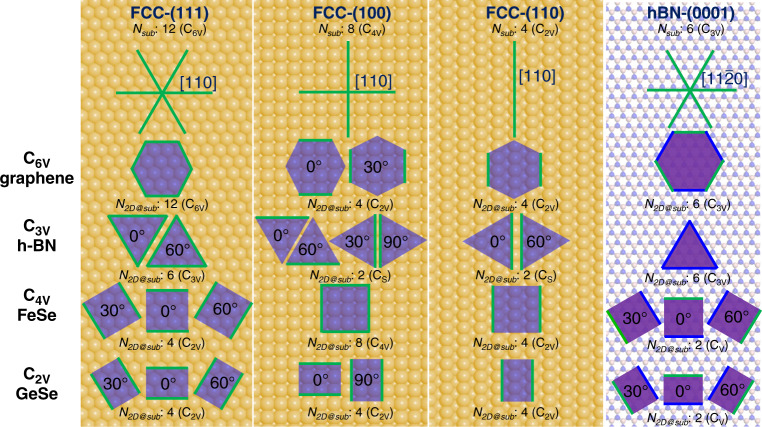
Alignment of single-crystalline 2D islands with various symmetries on low-index fcc surfaces and hBN (0001) surface. The 〈110〉 or 〈11${\bar{{2}}0}$〉 crystallographic orientations of substrates are denoted by green lines. 2D islands are represented by purple polygons. The symmetry groups of the 2D material, substrate, and the system of the 2D island on the substrate are provided. The number of different symmetry operations of the substrate and the whole system are *N*_sub_ and *N*_2D@sub_, respectively. The nitrogen and boron atoms are represented by blue and pink spheres, respectively. The relative anti-clockwise misorientation angles of grains with different orientations are given. Due to the C_3V_ symmetry of hBN, the edges of a 2D material, which are parallel to hBN 〈11${\bar{{2}}0}$〉 directions, have different local environments and not equivalent, and thus are denoted by blue and green lines, respectively.

Without loss of generality, we have used fcc(111), fcc(100), fcc(110), and hBN(0001) surfaces as different examples of a substrate with 6-, 4-, 2- and 3-fold symmetries to illustrate the alignment of various 2D materials on them. Figure 4 presents the various ways in which 2D materials with 2-, 3-, 4- and 6-fold symmetries are aligned on these substrates. From the figure, we can deduce that in order to keep the whole system with the highest symmetry, there are:

- 1, 2, 1 and 1 equivalent but different alignments for a 6-fold symmetric 2D material on 6-, 4-, 2- and 3-fold symmetric substrates;
- 2, 4, 2, and 1 equivalent but different alignments for a 3-fold symmetric 2D material on 6-, 4-, 2- and 3-fold symmetric substrates;
- 3, 1, 1 and 3 equivalent but different alignments for a 4-fold symmetric 2D material on 6-, 4-, 2- and 3-fold symmetric substrates;
- 3, 2, 1 and 3 equivalent but different alignments for a 2-fold symmetric 2D material on 6-, 4-, 2- and 3-fold symmetric substrates, respectively.

The number of equivalent but different alignments of various 2D materials on substrates of different symmetries are shown in Fig. 4 and these numbers are in perfect agreement with the symmetry analysis shown in Table 1. Besides the number of equivalent but different alignments of a 2D material on a substrate, Fig. 4 also gives the misalignment angles of equivalent islands of 2D materials. On the substrates with 6-, 4-, 3-, 2-fold symmetries, the misalignment angles are $\frac{i}{3}\pi ,\frac{i}{2}\pi ,\frac{{2i}}{3}\pi ,i\pi ,i = 1,2,3,$… respectively.

It is important to note that a high-symmetric edge of a 2D material along a high-symmetric direction of a substrate is critical for the above analysis. If the high-symmetric edge of the 2D material is along a low-symmetric direction of the substrate with mirror symmetry, the symmetry group G_2D@Sub_ will have no mirror symmetry and the least number of equivalent but different alignments of the 2D material will be 2, which makes orientational uniformity impossible.

### Comparison with experimental observations

We have summarized most of known experimental obversions on 2D materials epitaxial growth, including graphene growth on low-index Cu surfaces (Supplementary Table 1), hBN growth on low-index Cu surfaces (Supplementary Table 2), TMDCs growth on various low-index substrates, including Al_2_O_3_, Au, GaN, hBN (Supplementary Table 3), and various 2D material grown on different high-index substrates (Supplementary Table 4). It is interesting to note that there is a perfect agreement between the experimentally observed numbers of alignments and misalignment angles of 2D materials on these substrates with those predicted by our theoretical analysis. Hence, we believe that the epitaxial relationship is valid for the epitaxial growth of most 2D materials grown on different substrates.

### Strategies toward the epitaxial growth of various 2D single crystals

From Eq. (1), we can see that once the symmetry group of the substrate, G_Sub_, is a subgroup of the 2D material, G_2D_, or in other words

2 $${\mathrm{G}}_{{\mathrm{Sub}}} \subset {\mathrm{{G}}}_{2{\mathrm{D}}},$$

the symmetry group of the system G_2D@Sub_ could be same as G_Sub_. This means that there is only one most preferential alignment of the 2D material on the substrate and the orientational uniformity of a large number of islands of the 2D material is possible. As shown in Table 1 and Fig. 4, this can be applied to 2D materials with 6-fold symmetry, such as graphene on a 6-, 3- or 2-fold symmetric substrate, 2D materials with 4-fold  symmetric on a 4- or 2-fold symmetric substrate, 3-fold symmetricy 2D materials on a substrate with 3-fold symmetry, and a 2-fold symmetric 2D material on a 2-fold symmetric substrate. Up to now, the seamless stitching of well-aligned graphene islands have been realized on Cu(111) and Ge(110) surfaces, both of which are in accordance with the above described analysis^5–7,37,38^.

**Fig. 5:**
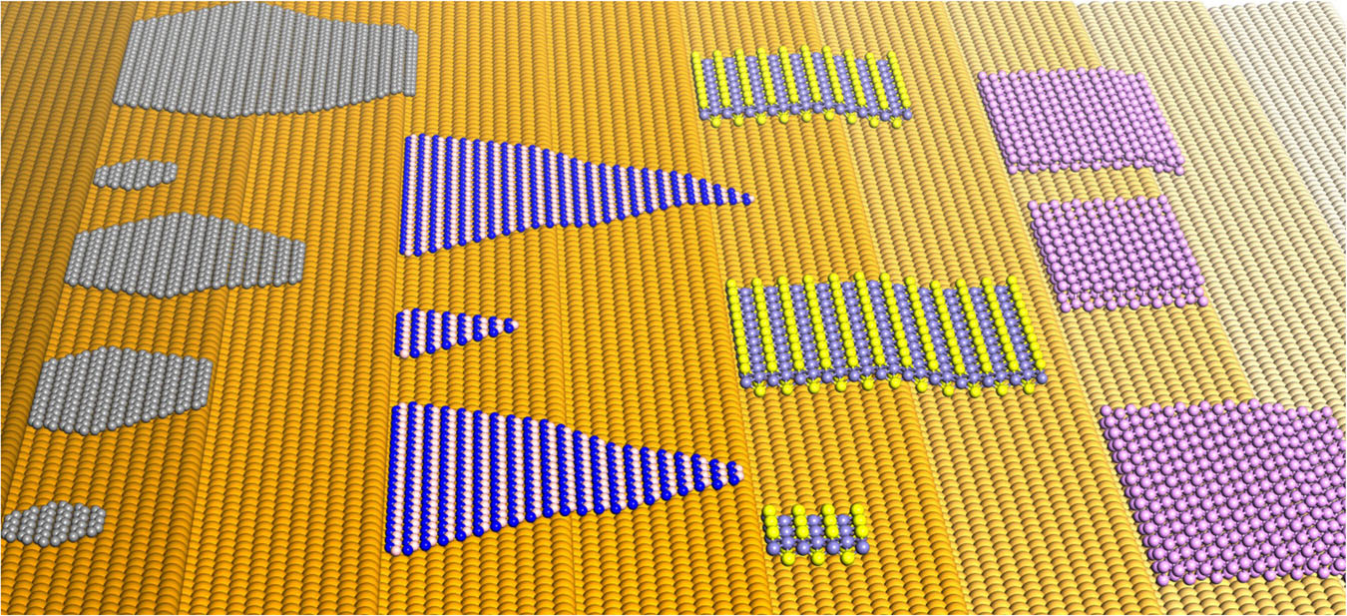
Schematics showing well-aligned 2D materials (from left to right are graphene, hBN, FeSe and black phosphorene, respectively.) on a vicinal fcc(111) surface. One edge of each island is passivated by a high-symmetric step edge of the substrate.

Among the thousands of known 2D materials, most of them have the 3-fold symmetry, such as the most explored hBN and TMDCs. As shown above, a substrate with 3-fold symmetry is expected to be suitable for the epitaxial growth of 2D materials with a three-fold symmetry, but it is difficult to find proper low-index substrates with 3-fold symmetry. Although some low-index substrates has the 3-fold symmetry, such as the Cu(111) surface and the Al_2_O_3_(0001) surface, the atoms of the top layer of the substrate generally have a higher symmetry, such as the atoms of the top Cu(111) layer have the 6-fold symmetry. So, for a C_3V_ 2D material on a C_3V_ substrate, there are generally two deep local minima in the formation profile which corresponds two high symmetric configurations, such as the 0˚ and 60˚ configurations of a WS_2_ on the hBN surface shown in Fig. 3d and Supplementary Fig. 10, and the 0˚ and 60˚ configurations of MoS_2_ on the Al_2_O_3_ (0001) surface in Fig. 3h of ref. ^34^ (please refer to Supplementary Fig. 11 for the configuration difference). Experimentally, anti-parallel TMDC islands on 3-fold symmetric substrates are generally seen and parallel aligned TMDC islands could be realized by precise control of the experimental condition (Supplementary Table 3)^17–21^. From Eq. (2), we can see that if the substrate has the C_1_ symmetry group, the condition for epitaxial growth can be satisfied for any 2D materials, implying that on a substrate with no symmetry, we may be able to achieve orientational uniformity for any 2D materials. In practice, substrates with very low symmetry could be a high-index surface or a vicinal surface of a low-index surface. As illustrated in Fig. 5, a high-index surface has a large number of low-index terraces connected by parallel step edges. These step edges of the substrate can serve as nucleation sites to initiate the growth of the 2D material. Furthermore, these step edges interact preferentially with an edge of the 2D material to promote the orientational uniformity of the 2D material. In this manner, the orientational uniformity of various 2D materials has been widely observed. As listed in Supplementary Table 4, epitaxial growth of well-aligned hBN islands have been observed on Cu(102), Cu(103), and vicinal Cu(110) surfaces^39,40^, where one of the three edges of the triangular hBN island is aligned along the step edge of the substrate. In addition, well-aligned WSe_2_ islands were also observed on Al_2_O_3_(0001) surface with step edges^22^. Recently, such a strategy has been used to grow wafer scale single-crystalline hBN on vicinal Cu(110) surface and Cu(111) surface with step egdes^8,9^, and centimeter scale single-crystalline MoS_2_ on an Au(111) surface with aligned step edges^10^. DFT calculations in these studies have shown that the weak interaction between the bulk of the 2D material and the substrate and/or the strong interaction between the edge of 2D material and step edge of the substrate lead to the favorable alignment of the 2D islands along the step edges of the substrate^8–10,39,40^. Since high-index surfaces can be easily obtained by miscutting a single crystal, we believe that this could be a general strategy for the synthesis of various 2D materials in the future.

## Discussion

We would like to note that the fact that a high symmetric direction of a 2D material prefers to align along a high symmetric direction of a substrate, which is revealed by our extensive DFT calculations in this study, is the foundation of our main conclusion. Otherwise, orientational uniformity of a 2D material on a substrate, even the symmetry group of the substrate is a subgroup of that of the 2D material, cannot be realized. Currently, most previous studies on the synthesis of large-sized single crystalline hBN and MoS_2_ employed vicinal (111) or (110) surfaces with parallel step edges^8–10^, where 2D materials tend to align along these step edges and the general study on the epitaxial 2D materials growth on various high index surface is very rare. Besides the vicinal surfaces which are close to one of the low-index surfaces, our study also predicts that the high-index surfaces that are largely deviated from all the low-index surfaces, such as the (123) surface of an fcc material, are also ideal for the epitaxial growth of large-scale single-crystalline 2D materials. Furthermore, our theory provides a principle to determine the alignment of a 2D material on any given substrates and, thus, it offers a strategy of synthesizing 2D materials with well-defined grain boundaries, for instance, polycrystalline graphene with grain boundaries of a 30^o^ misorientation angle can be synthesized on an fcc(100) surface, similar to the case of graphene growth on a liquid Cu surface^41^.

In conclusion, our study clearly demonstrates that the interplay of the symmetries of the 2D material and the substrate is critical for the epitaxial growth of 2D materials. Both theoretical analysis and experimental observations show that a high-symmetric direction of a 2D material tends to be aligned along a high-symmetric direction of a substrate, so that the 2D material–substrate system has the highest possible symmetry. Based on the symmetry analysis and the rules for the preferential alignment of a 2D material on a substrate, we established a library of the different possible alignments of various 2D materials on different substrates to serve as a guideline for experimental design. Furthermore, we theoretically proved that the epitaxial growth of a 2D single crystal can be realized only if the symmetry group of the substrate is a subgroup of that of the 2D material. To meet the requirement for single-crystalline 2D material growth, we propose using substrates with high-index surfaces which have lower symmetry to template the epitaxial growth of various 2D materials; this strategy has been successfully demonstrated (Nature 570, 91 (2019); Nature 579, 219 (2020); ACS nano 14, 5036 (2020)) and is in agreement with experimental observations. After submission of this manuscript, we have noticed that same strategy has been employed for the epitaxial synthesis of single-crystalline nanoribbons of TMDCs (Nat. Mater. DOI 10.1038/s41563-020-0795-4 (2020)), which further validated the proposed approach of synthesizing large area single-crystal 2D materials. Our study thus provides a theoretical foundation for the synthesis of wafer-scale 2D single crystals.

## Methods

### DFT calculations of edge binding energies

All DFT calculations were carried out via the Vienna ab initio simulation Package (VASP)^42,43^. The exchange-correlation effect was treated by the Perdew-Burke-Ernzerhof generalized gradient approximation (GGA)^44^. The interaction between valence electrons and ion cores was described by the projected augmented wave (PAW) method^45^ and the k-point mesh was sampled by a separation of 0.03 Å^−1^.

To calculate the binding energy of a graphene ZZ edge to Cu (111), (100), and (110) surfaces, Cu slabs consisting of three (111), (100), or (110) atomic layers were constructed to mimic the Cu substrates. Graphene nanoribbons along the ZZ direction, which were three hexagons wide and one of the two ZZ edges passivated by hydrogen, were constructed. Because of the incommensurate lattice constants between the graphene ZZ nanoribbon and the Cu substrates, only a small number of periodic structures can be constructed with a graphene ZZ nanoribbon adsorbed on low-index Cu surfaces along different directions, of which the number of atoms is not too large and can be handled by DFT calculations, as shown in Supplementary Figs. 1–3. The initial distance between the graphene ZZ nanoribbon and the Cu substrate is set to 3.06 Å, which was estimated from DFT-D2 calculations^46^, and it is the typical equilibrium distance between a graphene layer and the underlying Cu substrate surface. Structure optimization was conducted with the atomic positions of the lowest Cu atomic layer fixed. In addition, the vertical positions of carbon atoms that are passivated by hydrogen were also fixed during structure optimization. To eliminate the imaginary interaction between periodic images along the vertical direction, a vacuum layer with a 15 Å thickness was used to separate the Cu slabs. All the structures were relaxed until the force on each unfixed atom was <0.01 eV/Å, with an energy convergence of 10^−4^ eV.

In a similar manner, hBN nanoribbons along the ZZ direction were also constructed with their ZZB edges passivated by hydrogen. The structures with hBN ZZ ribbons adsorbed on low-index Cu substrate surfaces are shown in Supplementary Figs. 4 and 6. The distance between hBN nanoribbon and the Cu substrate is set to be 3.10 Å, which was obtained by optimizing a hBN sheet on Cu(111) surface by DFT-D2 calculations^46^. During structure optimization, the vertical positions of boron atoms passivated by hydrogen were fixed. In addition, the atomic positions of the lowest Cu atomic layer were also fixed.

The method similar to above calculations was also employed to calculate the binding energies between the graphene ZZ edge and Pt(111) surface. The calculated structures are shown in Supplementary Fig. 7.

The binding energy of a ZZ edge of graphene or hBN to the substrate is defined as

3 $$E_{\rm{{b}}} = \frac{{E_{{\rm{{total}}}} - E_{{\rm{{sub}}}} - E_{{\rm{{ribbon}}}}}}{l},$$

where *E*_ribbon_, *E*_sub_, and *E*_total_ are, respectively, the energies of the nanoribbon, the substrate and the total energy of the nanoribbon adsorbed on the substrate; *L* is the edge length of the nanoribbon.

To calculate the weak interaction between hBN wall and Cu(111) or Au(111) surface, a hBN layer was stacked to a Cu(111) or Au(111) slab consisting of three atomic layers under periodic boundary condition and with different alignment angles. The calculated structures are provided in Supplementary Figs. 8 and 9. During structure optimization, the bottom atomic layer of the metal slab was fixed. The binding energy between the hBN wall and the metal substrate is defined as

4 $$E_{\rm{{b}}} = \frac{{E_{{\rm{{total}}}} - E_{{\rm{{hBN}}}} - E_{{\rm{{sub}}}}}}{{N_{{\rm{{BN}}}}}},$$

where *E*_total_, *E*_hBN_, and *E*_sub_ are the energies of the whole system, the hBN layer and the substrate, respectively. *N*_BN_ is the number of BN pairs of the hBN layer in the unit cell of the whole system.

To calculate the weak interaction between WS_2_ and hBN layer, a triangular WS_2_ cluster consisting of 60 S atoms and 27 W atoms was placed on a hBN layer with different orientations (Supplementary Fig. 10). Because the edges of the WS_2_ cluster are passivated by S and the hBN layer is chemically inert, the interaction between the WS_2_ cluster and the hBN layer should be dominated by the WS_2_ wall and the hBN layer. The binding energy between the WS_2_ cluster and the hBN layer is defined as

5 $$E_{\rm{{b}}} = \frac{{E_{{\rm{{total}}}} - E_{{\rm{{hBN}}}} - E_{{\rm{{WS}}}_2}}}{{N_{\rm{{W}}}}},$$

where *E*_total_, *E*_hBN_, and *E*_WS2_ are the energies of the whole system, the hBN layer and the WS_2_ cluster, respectively. *N*_W_ is the number of W atoms in the WS_2_ cluster.

## Supplementary information

Supplementary Information

## Data Availability

The data that support the findings of this study are available from the corresponding authors on reasonable request.

## References

[1] Geim AK, Novoselov KS (2007). The rise of graphene. Nat. Mater..

[2] Watanabe K, Taniguchi T, Kanda H (2004). Direct-bandgap properties and evidence for ultraviolet lasing of hexagonal boron nitride single crystal. Nat. Mater..

[3] Zhou J (2018). A library of atomically thin metal chalcogenides. Nature.

[4] Song C-L (2011). Molecular-beam epitaxy and robust superconductivity of stoichiometric FeSe crystalline films on bilayer graphene. Phys. Rev. B.

[5] Xu X (2017). Ultrafast epitaxial growth of metre-sized single-crystal graphene on industrial Cu foil. Sci. Bull..

[6] Deng B (2017). Wrinkle-free single-crystal graphene wafer grown on strain-engineered substrates. ACS Nano.

[7] Nguyen VL (2015). Seamless stitching of graphene domains on polished copper (111) foil. Adv. Mater..

[8] Wang L (2019). Epitaxial growth of a 100-square-centimetre single-crystal hexagonal boron nitride monolayer on copper. Nature.

[9] Chen T-A (2020). Wafer-scale single-crystal hexagonal boron nitride monolayers on Cu (111). Nature.

[10] Yang P (2020). Epitaxial growth of centimeter-scale single-crystal MoS2 monolayer on Au (111). ACS Nano.

[11] Murdock AT (2013). Controlling the orientation, edge geometry, and thickness of chemical vapor deposition graphene. ACS Nano.

[12] Ogawa Y (2012). Domain structure and boundary in single-layer graphene grown on Cu(111) and Cu(100) films. J. Phys. Chem. Lett..

[13] Wood GE (2015). van der Waals epitaxy of monolayer hexagonal boron nitride on copper foil: growth, crystallography and electronic band structure. 2D Mater..

[14] Song X (2015). Chemical vapor deposition growth of large-scale hexagonal boron nitride with controllable orientation. Nano Res..

[15] Uchida Y, Iwaizako T, Mizuno S, Tsuji M, Ago H (2017). Epitaxial chemical vapour deposition growth of monolayer hexagonal boron nitride on a Cu(111)/sapphire substrate. Phys. Chem. Chem. Phys..

[16] Grønborg SS (2015). Synthesis of epitaxial single-layer MoS2 on Au(111). Langmuir.

[17] Zhang X (2018). Diffusion-controlled epitaxy of large area coalesced WSe2 monolayers on sapphire. Nano Lett..

[18] Aljarb A (2017). Substrate lattice-guided seed formation controls the orientation of 2D transition-metal dichalcogenides. ACS Nano.

[19] Dumcenco D (2015). Large-area epitaxial monolayer MoS2. ACS Nano.

[20] Ruzmetov D (2016). Vertical 2D/3D semiconductor heterostructures based on epitaxial molybdenum disulfide and gallium nitride. ACS Nano.

[21] Lee JS (2018). Wafer-scale single-crystal hexagonal boron nitride film via self-collimated grain formation. Science.

[22] Chen L (2015). Step-edge-guided nucleation and growth of aligned WSe2 on sapphire via a layer-over-layer growth mode. ACS Nano.

[23] Dong J, Zhang L, Ding F (2019). Kinetics of graphene and 2D materials growth. Adv. Mater..

[24] Ma T (2013). Edge-controlled growth and kinetics of single-crystal graphene domains by chemical vapor deposition. Proc. Natl Acad. Sci. USA.

[25] Sekerka RF (2005). Equilibrium and growth shapes of crystals: how do they differ and why should we care?. Cryst. Res. Technol..

[26] Zhang Z, Liu Y, Yang Y, Yakobson BI (2016). Growth mechanism and morphology of hexagonal boron nitride. Nano Lett..

[27] Stehle YY (2017). Anisotropic etching of hexagonal boron nitride and graphene: question of edge terminations. Nano Lett..

[28] Tay RY (2016). Synthesis of aligned symmetrical multifaceted monolayer hexagonal boron nitride single crystals on resolidified copper. Nanoscale.

[29] Yuan Q, Yakobson BI, Ding F (2014). Edge-catalyst wetting and orientation control of graphene growth by chemical vapor deposition growth. J. Phys. Chem. Lett..

[30] Zhang X, Xu Z, Hui L, Xin J, Ding F (2012). How the orientation of graphene is determined during chemical vapor deposition growth. J. Phys. Chem. Lett..

[31] Dong J (2017). Formation mechanism of overlapping grain boundaries in graphene chemical vapor deposition growth. Chem. Sci..

[32] Ren X (2019). Grain boundaries in chemical-vapor-deposited atomically thin hexagonal boron nitride. Phys. Rev. Mater..

[33] Sang X (2018). In situ edge engineering in two-dimensional transition metal dichalcogenides. Nat. Commun..

[34] Shu H, Chen X, Ding F (2014). The edge termination controlled kinetics in graphene chemical vapor deposition growth. Chem. Sci..

[35] Dong J, Zhang L, Zhang K, Ding F (2018). How graphene crosses a grain boundary on the catalyst surface during chemical vapour deposition growth. Nanoscale.

[36] Ji Q (2015). Unravelling orientation distribution and merging behavior of monolayer MoS2 domains on sapphire. Nano Lett..

[37] Jin S (2018). Colossal grain growth yields single-crystal metal foils by contact-free annealing. Science.

[38] Lee J-H (2014). Wafer-scale growth of single-crystal monolayer graphene on reusable hydrogen-terminated germanium. Science.

[39] Li J (2016). Growth of polar hexagonal boron nitride monolayer on nonpolar copper with unique orientation. Small.

[40] Wang S (2019). Catalyst-selective growth of single-orientation hexagonal boron nitride toward high-performance atomically thin electric barriers. Adv. Mater..

[41] Dong J, Geng D, Liu F, Ding F (2019). Formation of twinned graphene polycrystals. Angew. Chem. Int. Ed..

[42] Kresse G, Hafner J (1993). Ab initio molecular dynamics for open-shell transition metals. Phys. Rev. B.

[43] Kresse G, Furthmüller J (1996). Efficiency of ab-initio total energy calculations for metals and semiconductors using a plane-wave basis set. Comput. Mater. Sci..

[44] Perdew JP, Burke K, Ernzerhof M (1996). Generalized gradient approximation made simple. Phys. Rev. Lett..

[45] Kresse G, Joubert D (1999). From ultrasoft pseudopotentials to the projector augmented-wave method. Phys. Rev. B.

[46] Grimme S (2006). Semiempirical GGA-type density functional constructed with a long-range dispersion correction. J. Comput. Chem..

