# Supplementary material for: The epitaxy of 2D materials growth
Source: Nat Commun. 2020 Nov 17;11:5862. doi: 10.1038/s41467-020-19752-3 (PMC7672100; doi:10.1038/s41467-020-19752-3)
Supplement: Supplementary file 1 — Supplementary Information [file 41467_2020_19752_MOESM1_ESM.pdf]

Supplementary information for:

The epitaxy of 2D materials growth

Jichen Dong[1], *Leining Zhang*[1,2], *Xinyue Dai*[1,3], *Feng Ding*[1,2,*]

[1] Centre for Multidimensional Carbon Materials, Institute for Basic Science, Ulsan 44919, Korea
[2] School of Materials Science and Engineering, Ulsan National Institute of Science and Technology, Ulsan 44919, Korea
[3] School of Materials Science and Engineering, Shandong University, Jinan 250061, China

* Email: f.ding@unist.ac.kr

Contents

1. **Density functional theory calculation details**
2. **Proof of Eqn. 1 in the main text**
3. **Experimental observation of alignment of 2D materials on various substrates**

1 Density functional theory calculation details

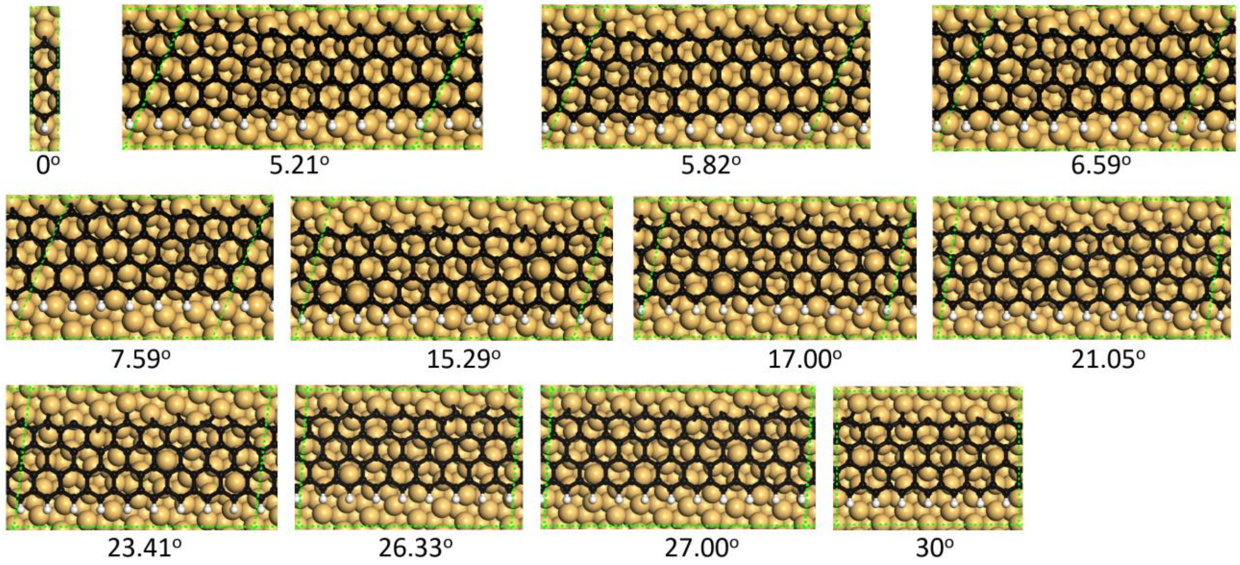

Supplementary Figure 1 Optimized structures for calculating the binding energy of a graphene ZZ edge to the Cu(111) surface. The angle between the graphene ZZ edge and the <110> crystallographic direction of the Cu substrate is given for each structure. The unit cell of each structure is denoted by green dashed rhomb.

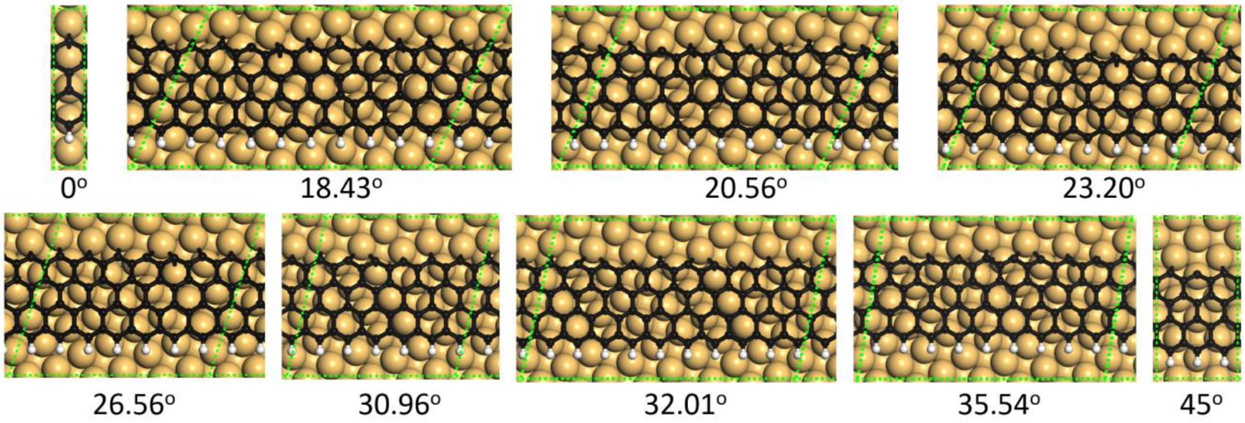

Supplementary Figure 2 Optimized structures for calculating the binding energy of graphene ZZ edge to Cu(100) surface. The angle between graphene ZZ edge and the <110> crystallographic direction of the Cu substrate is given for each structure. The unit cell of each structure is denoted by green dashed rhomb.

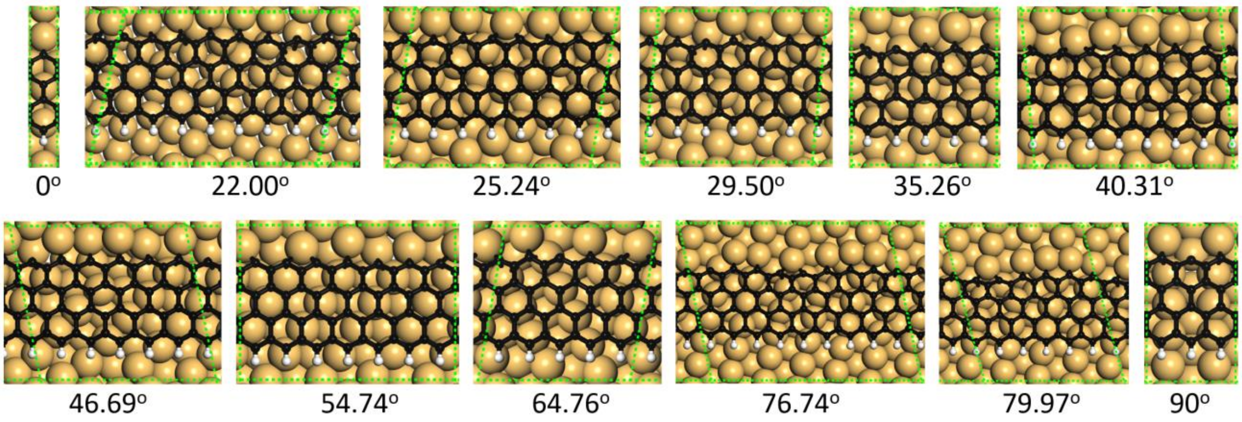

Supplementary Figure 3 Optimized structures for calculating the binding energy of graphene ZZ edge to Cu(110) surface. The angle between the graphene ZZ edge and the <110> crystallographic direction of the Cu substrate is given for each structure. The unit cell of each structure is denoted by green dashed rhomb.

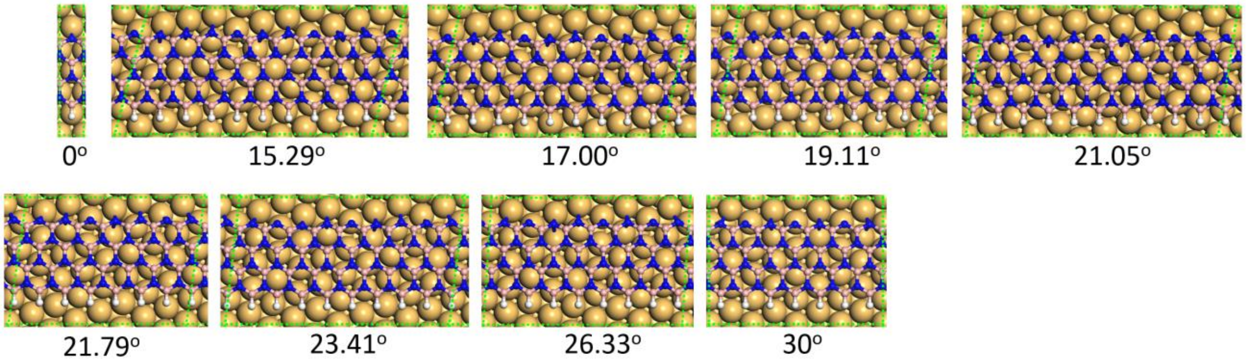

Supplementary Figure 4 Optimized structures for calculating the binding energy of hBN ZZN edge to Cu(111) surface. The angle between the hBN ZZ edge and the <110> crystallographic direction of the Cu substrate is given for each structure. The unit cell of each structure is denoted by green dashed rhomb.

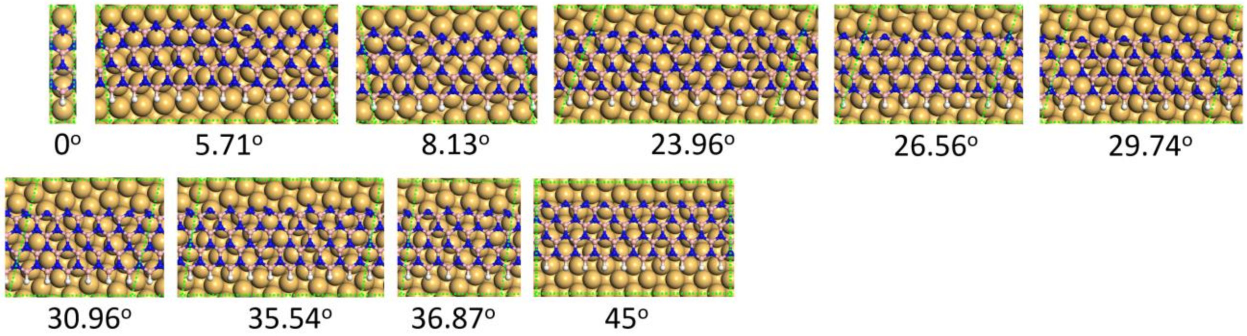

Supplementary Figure 5 Optimized structures for calculating the binding energy of hBN ZZN edge to Cu(100) surface. The angle between the hBN ZZ edge and the <110> crystallographic direction of the Cu substrate is given for each structure. The unit cell of each structure is denoted by green dashed rhomb.

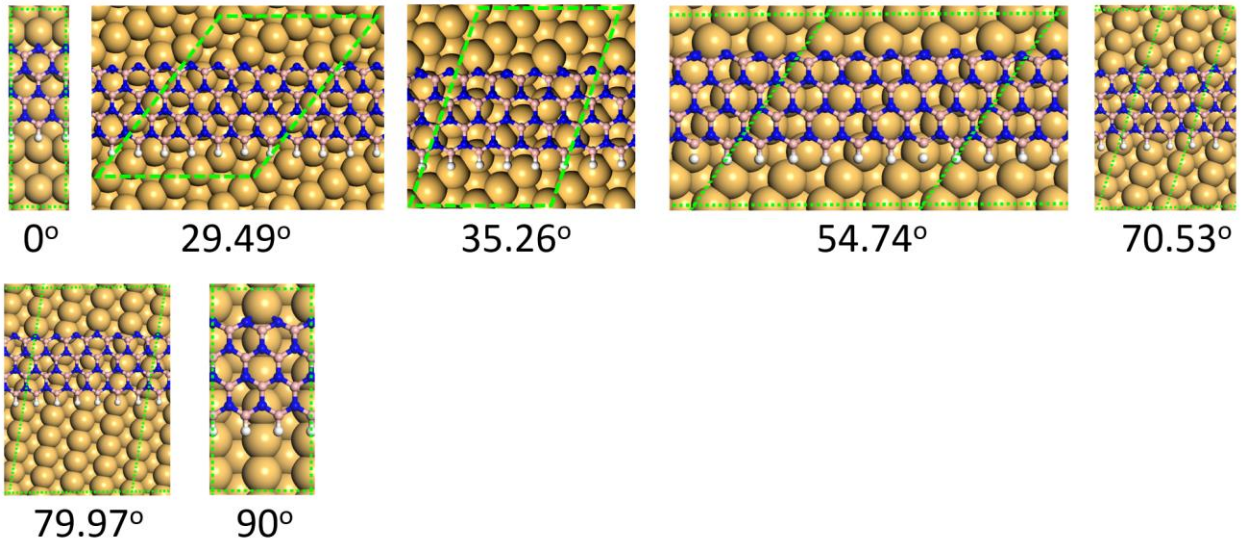

Supplementary Figure 6 Optimized structures for calculating the binding energy of hBN ZZN edge to Cu(110) surface. The angle between the hBN ZZ edge and the <110> crystallographic direction of the Cu substrate is given for each structure. The unit cell of each structure is denoted by green dashed rhomb.

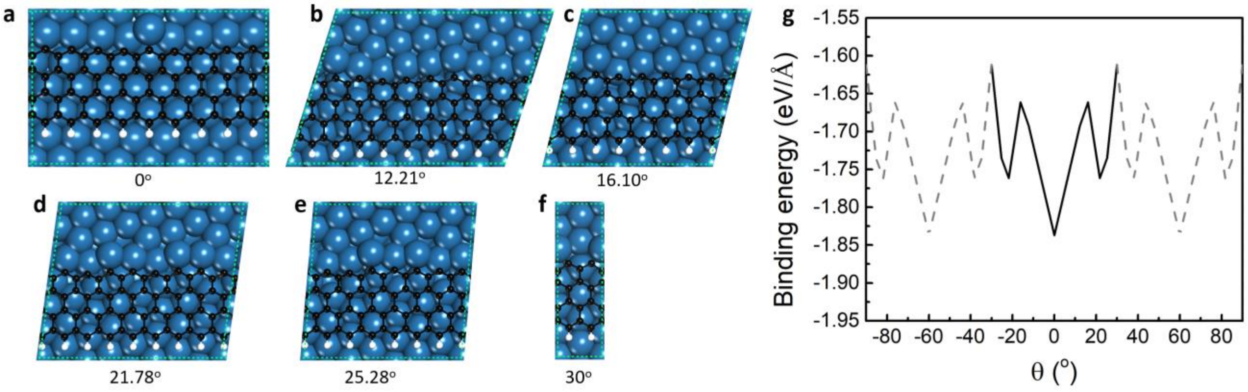

Supplementary Figure 7 Optimized structures for calculating the binding energies of graphene ZZ edge to Pt(111) surface (a-f). The angle between the graphene ZZ edge and the <110> crystallographic direction of the Pt substrate is given for each structure. The unit cell of each structure is denoted by green dashed rhomb. (g) shows the binding energy profile of the graphene ZZ edge to the Pt(111) substrate as a function of the alignment angle.

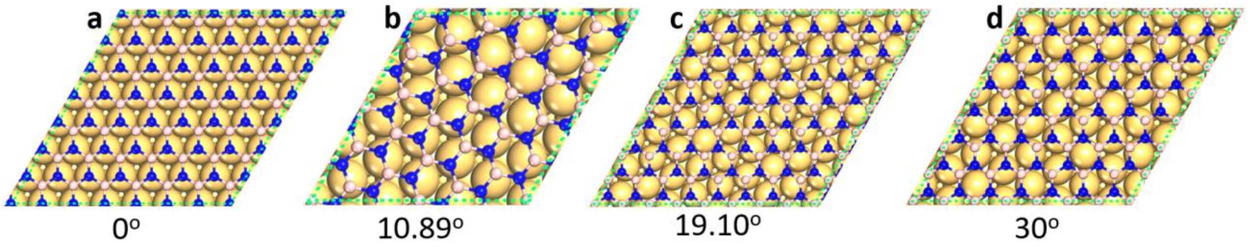

Supplementary Figure 8 Optimized structures for calculating the interaction between hBN bulk and Cu(111) surface (a-d). The angles between the ZZ direction of hBN and the Cu<110> direction are provided. The unit cell of each structure is denoted by green dashed rhomb.

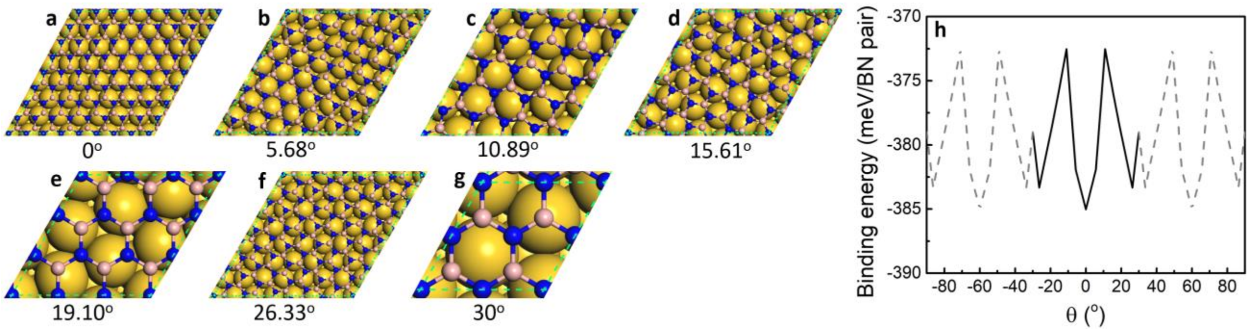

Supplementary Figure 9 Optimized structures for calculating the interaction between hBN bulk and Au(111) surface (a-g). The angle between the hBN ZZ direction and the <110> crystallographic direction of the Au substrate is given for each structure. The unit cell of each structure is denoted by green dashed rhomb. (h) shows the binding energy profile of the hBN wall to the Au(111) substrate as a function of the alignment angle.

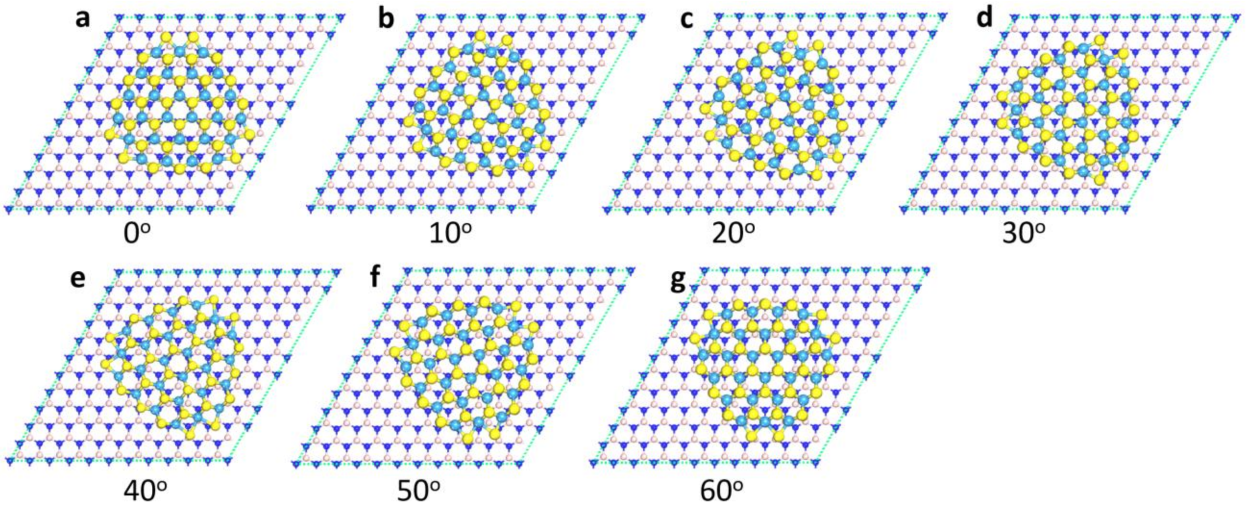

Supplementary Figure 10 Optimized structures for calculating the weak interaction between a triangular WS_2 cluster and hBN surface (a-g). The angle between the WS_2 ZZ direction and the hBN ZZ direction is given for each structure. The unit cell of each structure is denoted by green dashed rhomb.

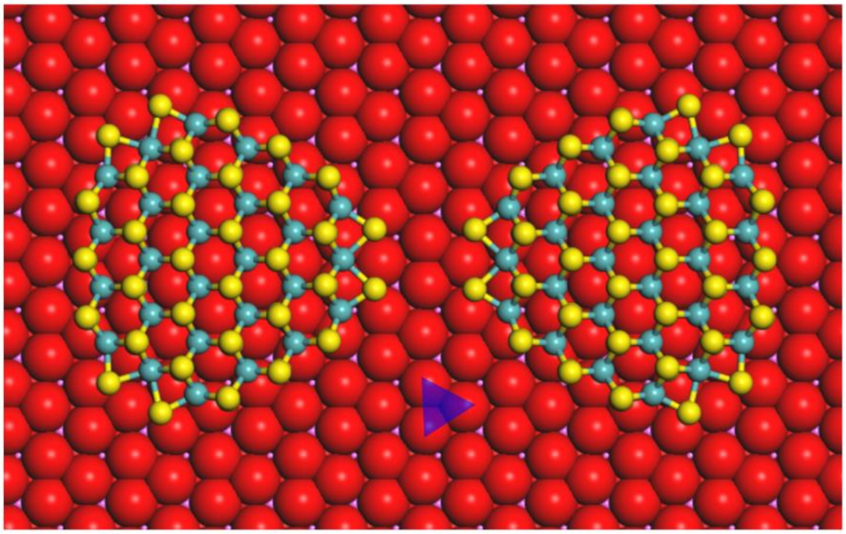

Supplementary Figure 11 Atomic model showing the configuration difference between two anti-parallel MoS_2 clusters on the oxygen terminated Al_2O_3 (0001) surface. Because the oxygen terminated Al_2O_3 (0001) surface is 3-fold symmetric, which is denoted by the blue triangle, the two anti-parallel MoS_2 clusters are actually non-equivalent. The oxygen atoms at the top atomic layer of the Al_2O_3 (0001) surface are represented by red large balls.

2 Proof of Eqn. 1 in the main text

Eqn. 1 is derived from the Lagrange's theorem of the group theory.

It is clear that the symmetry group of the system of a 2D material on a substrate, $G_{2D@Sub}$, must be a subgroup of the symmetric group of the substrate, G_{Sub} because any symmetric operation of $G_{2D@Sub}$ will not change the substrate. From the Lagrange's theorem[1], we have the following relationship between the order of G_{Sub} (the number of nonequivalent symmetry operations of G_{Sub}) and that of its subgroup, $G_{2D@Sub}$:

$$|G_{sub}| = [G_{sub}: G_{2D@sub}]|G_{2D@sub}|, \quad \text{(S1)}$$

where $|G_{sub}|$ and $|G_{2D@sub}|$ are the orders of G_{Sub} and $G_{2D@Sub}$, respectively. $N = [G_{sub}: G_{2D@sub}]$ is the number of nonequivalent left cosets of $G_{2D@Sub}$ in G_{Sub}. According to the Lagrange's theorem, G_{Sub} can be constructed as:

$$G_{sub} = g_1 G_{2D@sub} \cup g_2 G_{2D@sub} \cup \cdots \cup g_i G_{2D@sub} \cup \cdots \cup g_N G_{2D@sub}, \quad \text{(S2)}$$

where $g_i \in G_{sub}$, $g_1 = E$, and $g_i G_{2D@sub} \neq g_j G_{2D@sub}$ unless $i = j$.

Except $g_1 G_{2D@sub} = G_{2D@sub}$, applying all symmetric operations of a left corset, $g_i G_{2D@sub}$, to the system of 2D@Sub will change the alignment of the 2D material to an equivalent but different direction. So, the number of equivalent but different directions of a 2D material on a substrate is the number of nonequivalent left cosets of $G_{2D@Sub}$ in G_{Sub}:

$$N = [G_{sub}: G_{2D@sub}] = |G_{sub}|/|G_{2D/sub}|. \quad \text{(S3)}$$

3 Experimental observation of various 2D materials epitaxial growth on various substrates and the comparison with theoretical predictions.

Supplementary Table 1 shows the experimentally observed alignments of graphene on various low-index Cu surfaces. In perfect agreement with above theoretical predictions, the number of non-alignments of graphene islands on Cu(111), Cu(100) and Cu(110) are 1, 2, 1 respectively, which have been clearly seen in a large number of experiments. The misalignment of the two types of graphene domains on the Cu(100) surface with a 30 degree misorientation angle has been observed [2,3]. Besides, the alignment of graphene ZZ edge along the Cu<110> direction has also been confirmed [2,3,4].

Supplementary Table 2 shows the experimental observations of hBN on various Cu surfaces. As the theoretical analyses shown in Fig. 4 in the main text, antiparallelly aligned triangular hBN grains on both Cu(111) and Cu(110) surfaces and the four different orientations of triangular hBN grains on Cu(100)

surface have been clearly seen[5,6,7,8]. The alignment of ZZN edge of hBN domains along the <110> direction of the substrate has been shown in many experimental observations as well[9,10,11].

Supplementary Table 3 presents a summary of the epitaxial grown TMDCs on various substrates, including both metallic Au surface and the nonmetallic substrates. Most TMDCs have a C_{3V} symmetry as hBN and the predicted alignment of a TMDC on a substrate are exactly same as that of hBN. Antiparallel domains of TMDCs on the six-fold symmetric Au(111)[9], Al_2O_3 [10,11,12]and GaN(0001) surfaces [13] are clearly seen; the unidirectionally aligned WS_2 islands were observed to grown on a C_{3V} symmetric hBN surface [14].

The unidirectional alignment of hBN on high index Cu surface have been observed (Supplementary Table 4). Very recently, Liu and Chen *et al.* successfully synthesized large-sized single crystalline h-BN on a vicinal Cu(110) and Cu(111) surface, respectively, where all the triangular h-BN grains are unidirectionally aligned [15,16]. Guo and Wang at al. also observed unidirectionally aligned hBN grains grown on many vicinal Cu(110) surfaces [17,18]. Moreover, unidirectionally aligned WSe_2 islands have also been observed on a vicinal Al_2O_3(0001) surface with parallel step edges [19]. Centimeter scale single crystalline MoS_2 has been obtained by seamless coalescence of unidirectionally aligned MoS_2 grains grown on a vicinal Au(111) surface[20]. These studies strongly validate our theoretical analysis that using substrates with a high-index surface and a low symmetry might be a new direction on synthesizing large-sized single crystalline 2D materials.

Supplementary Table 1 Alignment of graphene on low-index Cu surfaces. N represents the number of alignments and θ represents the misorientation angle of graphene grains, respectively. ZZ represents the zigzag edge of graphene. In which, ZZ//Cu(110) represents the zigzag edge of graphene parallel to a Cu<110> direction of the Cu surface and etc.

**2D Material**	**Substrate type**	**Experimental Observations**				**Ref.**	**Theoretical Predictions**		
		**Graphene Alignment**	**N**	**θ**	**Figures**		**Graphene Alignment**	**N**	**θ**
Graphene (C_{6V})	Cu(111) (C_{6V})	ZZ//Cu<110>	1	0°	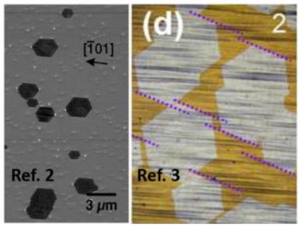	2,3,4	ZZ//Cu<110>	1	0°
Graphene (C_{6V})	Cu(110) (C_{2V})	ZZ//Cu<110>	1	0°	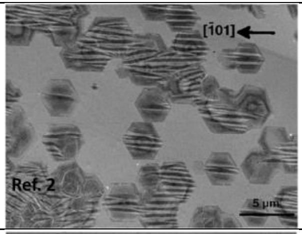	2	ZZ//Cu<110>	1	0°
Graphene (C_{6V})	Cu(100) (C_{4V})	ZZ//Cu<110>	2	30°	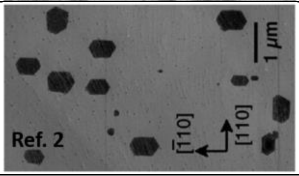	2,3	ZZ//Cu<110>	2	30°

For scanning electron microscopy (SEM) images in the 2, 3 and 4 rows, adapted with permission from Ref. 2. Copyright (2013) American Chemical Society. For the optical microscope (OM) image in the second row, reprinted from Ref. 3, Copyright (2017), with permission from Elsevier.

Supplementary Table 2 Alignment of hBN on low-index Cu surfaces. N represents the number of alignments and θ represents the misorientation angle of hBN grains, respectively. ZZ represents the zigzag edge of hBN.

**2D Material**	**Substrate type**	**Experimental Observation**				**Ref.**	**Theoretical Prediction**		
		**hBN Alignment**	**N**	**θ**	**Figures**		**hBN Alignment**	**N**	**θ**
hBN (C_{3v})	Cu(111) (C_{6v})	ZZ //Cu<110>	2	60°	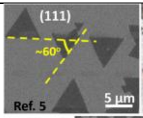 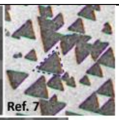 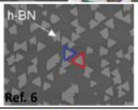	5,6,7	ZZ //Cu<110>	2	60°
hBN (C_{3v})	Cu(110) (C_{2v})	N/A	2	60°	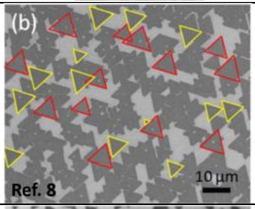	8	ZZ //Cu<110>	2	60°
hBN (C_{3v})	Cu(100) (C_{4v})	ZZ //Cu<110>	4	30°, 60° and 90°	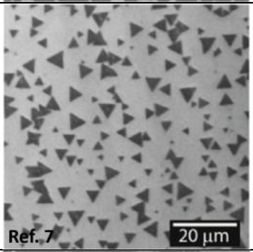	7	ZZ //Cu<110>	4	30°, 60° and 90°
hBN (C_{3v})	Cu(100) (C_{4v})	N/A	4	30°, 60° and 90°	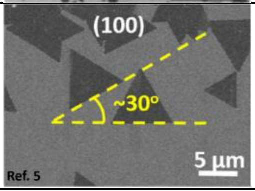	5	ZZ //Cu<110>	4	30°, 60° and 90°

For the SEM images including the upper left one in row 2 and the one in row 5, reprinted with permission from Ref. 5, Spring Nature, Copyright (2015). For the lower SEM image in row 2, reproduced from Ref. 6 with permission from the PCCP Owner Societies. For the upper right SEM image in row 2 and the SEM image in row 4, reprinted with permission from Ref. 7, IOP Publishing Ltd, Copyright (2015). For the SEM image in row 3, reproduced from Ref. 8 with permission from The Royal Society of Chemistry.

Supplementary Table 3 Alignment of TMDCs on low-index substrate surfaces. N represents the number of alignments and θ represents the misorientation angle of TMDC grains, respectively. ZZ represents the zigzag edge of TMDCs.

2D Material	Substrate type	Experimental Observation				Refs.	Theoretical Prediction		
		TMDC Alignment	N	θ	Figures		TMDC Alignment	N	θ
MoS_2 (D_{3h})	Au(111) (C_{6v})	ZZ// Au<110>	2	60°	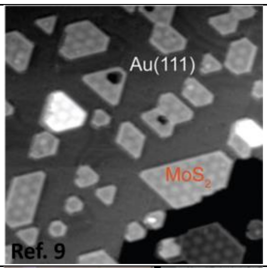	9	ZZ//Au<110>	2	60°
MoS_2 (D_{3h})	Al_2O_3(0001) (C_{3v})	N/A	2	60°	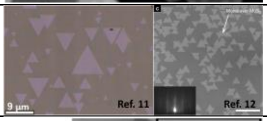	11,12	ZZ// $Al_2O_3\langle 11\bar{2}0\rangle$	2	60°
WSe_2 (D_{3h})	Al_2O_3(0001) (C_{3v})	ZZ// $Al_2O_3\langle 11\bar{2}0\rangle$	2	60°	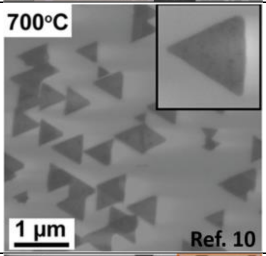	10	ZZ// $Al_2O_3\langle 11\bar{2}0\rangle$	2	60°
MoS_2 (D_{3h})	GaN(0001) (C_{6v})	ZZ// GaN$\langle 11\bar{2}0\rangle$	2	60°	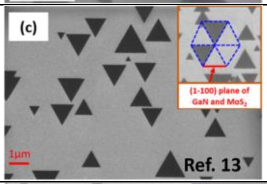	13	ZZ// GaN$\langle 11\bar{2}0\rangle$	2	60°
WS_2 (D_{3h})	hBN(0001) (C_{3v})	ZZ// hBN$\langle 11\bar{2}0\rangle$	1	0°	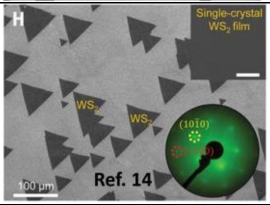	14	ZZ// hBN$\langle 11\bar{2}0\rangle$	1	0°

For the SEM image in row 2, adapted with permission from Ref. 9, Copyright (2015) American Chemical Society. For the OM image in row 3, adapted with permission from Ref. 11, Copyright (2017) American Chemical Society. For the SEM image in row 3, adapted with permission from Ref. 12, Copyright (2015) American Chemical Society. For the SEM image in row 4, adapted with permission from Ref. 10, Copyright (2015) American Chemical Society. For the SEM image in row 5, adapted with permission from Ref. 13, Copyright (2015) American Chemical Society. For the SEM image in row 6, From Ref. 14, Reprinted with permission from AAAS.

Supplementary Table 4 Alignment of 2D materials on high-index substrate surfaces. N represents the number of alignments and θ represents the misorientation angle of 2D grains, respectively. ZZ represents the zigzag edge of 2D grains.

2D Material	Substrate type	Experimental Observation				Refs.	Theoretical Prediction		
		2D materials Alignment	N	θ	Figures		2D materials Alignment	N	θ
hBN (C_{3v})	Vicinal Cu(110) (C_1 or C_s)	ZZ // Cu step edges	1	0°	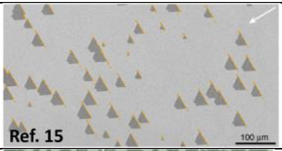	15	ZZ // Cu step edges	1	0°
hBN (C_{3v})	Cu(14 1 15) (C_1)	ZZ // Cu step edges	1	0°	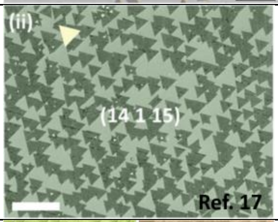	17	ZZ // Cu step edges	1	0°
hBN (C_{3v})	Cu(1 0 2) Cu(1 0 3) (C_1)	ZZ // Cu step edges	1	0°	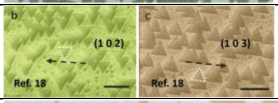	18	ZZ // Cu step edges	1	0°
hBN (C_{3v})	Cu(111) with step edges (C_1 or C_s)	ZZ // Cu step edges	1	0°	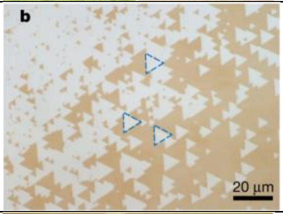	16	ZZ // Cu step edges	1	0°
WSe_2 (D_{3h})	Vicinal Al_2O_3(0001) (C_1 or C_s)	ZZ // Al_2O_3 step edges	1	0°	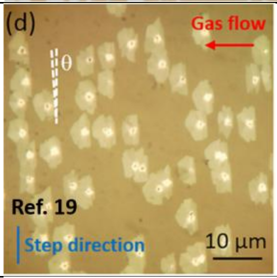	19	ZZ // Al_2O_3 step edges	1	0°
MoS_2 (D_{3h})	Vicinal Au(111) (C_1 or C_s)	ZZ // Au step edges	1	0°	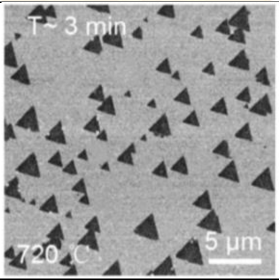	20	ZZ // Au step edges	1	0°

For the SEM image in row 2, reprinted with permission from Ref. 15, Spring Nature, Copyright (2019). For the SEM image in row 3, reprinted with permission from Ref. 17, John Wiley and Sons, Copyright (2019). For the SEM images in row 4, reprinted with permission from Ref. 18, John Wiley and Sons, Copyright (2016). For the OM image in row 5, reprinted with permission from Ref. 16, Spring Nature, Copyright (2020). For the OM image in row 6, adapted with permission from Ref. 19, Copyright (2015) American Chemical Society. For the OM image in row 7, adapted with permission from Ref. 20, Copyright (2020) American Chemical Society.

It should be noted that only the top atomic layer is considered for determining the symmetries of the substrates, because the 2D material mainly interacts with the top atomic layer of the substrate, as has exemplified in Fig. 2 in the main text.

Supplementary References

1 Hamermesh M. Group Theory and Its Application to Physical Problems. (Courier Corporation, 1989)

2 Murdock, A. T. *et al.* Controlling the Orientation, Edge Geometry, and Thickness of Chemical Vapor Deposition Graphene. *ACS Nano* **7**, 1351-1359 (2013).

3 Ogawa, Y. *et al.* Domain Structure and Boundary in Single-Layer Graphene Grown on Cu(111) and Cu(100) Films. *J. Phys. Chem. Lett.* **3**, 219-226 (2012).

4 Xu, X. *et al.* Ultrafast epitaxial growth of metre-sized single-crystal graphene on industrial Cu foil. *Sci. Bull.* **62**, 1074-1080 (2017).

5 Song, X. *et al.* Chemical vapor deposition growth of large-scale hexagonal boron nitride with controllable orientation. *Nano Res.* **8**, 3164-3176 (2015).

6 Uchida, Y., Iwaizako, T., Mizuno, S., Tsuji, M. & Ago, H. Epitaxial chemical vapour deposition growth of monolayer hexagonal boron nitride on a Cu(111)/sapphire substrate. *Phys. Chem. Chem. Phys.* **19**, 8230-8235 (2017).

7 Wood, G. E. *et al.* van der Waals epitaxy of monolayer hexagonal boron nitride on copper foil: growth, crystallography and electronic band structure. *2D Mater.* **2**, 025003 (2015).

8 Tay, R. Y. *et al.* Synthesis of aligned symmetrical multifaceted monolayer hexagonal boron nitride single crystals on resolidified copper. *Nanoscale* **8**, 2434-2444 (2016).

9 Grønborg, S. S. *et al.* Synthesis of Epitaxial Single-Layer MoS2 on Au(111). *Langmuir* **31**, 9700-9706 (2015).

10 Zhang, X. *et al.* Diffusion-Controlled Epitaxy of Large Area Coalesced WSe2 Monolayers on Sapphire. *Nano Lett.* **18**, 1049-1056 (2018).

11 Aljarb, A. *et al.* Substrate Lattice-Guided Seed Formation Controls the Orientation of 2D Transition-Metal Dichalcogenides. *ACS Nano* **11**, 9215-9222 (2017).

12 Dumcenco, D. *et al.* Large-Area Epitaxial Monolayer MoS2. *ACS Nano* **9**, 4611-4620 (2015).

13 Ruzmetov, D. *et al.* Vertical 2D/3D Semiconductor Heterostructures Based on Epitaxial Molybdenum Disulfide and Gallium Nitride. *ACS Nano* **10**, 3580-3588 (2016).

14 Lee, J. S. *et al.* Wafer-scale single-crystal hexagonal boron nitride film via self-collimated grain formation. *Science* **362**, 817-821 (2018).

15 Wang, L. *et al*. Epitaxial growth of a 100-square-centimetre single-crystal hexagonal boron nitride monolayer on copper. *Nature* **570**, 91-95 (2019).

16 Chen, T.-A. *et al.* Wafer-scale single-crystal hexagonal boron nitride monolayers on Cu (111). *Nature* **579**, 219-223 (2020).

17 Wang, S. *et al.* Catalyst-Selective Growth of Single-Orientation Hexagonal Boron Nitride toward High-Performance Atomically Thin Electric Barriers. *Adv. Mater.* **31**, 1900880 (2019).

18 Li, J. *et al.* Growth of Polar Hexagonal Boron Nitride Monolayer on Nonpolar Copper with Unique Orientation. *Small* **12**, 3645-3650 (2016).

19 Chen, L. *et al.* Step-Edge-Guided Nucleation and Growth of Aligned WSe2 on Sapphire via a Layer-over-Layer Growth Mode. *ACS Nano* **9**, 8368-8375 (2015).

20 Yang, P. *et al.* Epitaxial Growth of Centimeter-Scale Single-Crystal MoS2 Monolayer on Au (111). *ACS Nano* (2020).
